# Headache

**Published:** 1846-03

**Authors:** 


*Headache.*—Dr. McCready relates cases of violent headache relieved by the use of the saturated *Tincture of Aconite* in doses of four or five drops taken internally.—*N. York Jour.*

